# Physical activity, resilience, emotions, moods, and weight control, during the COVID-19 global crisis

**DOI:** 10.1186/s13584-021-00473-x

**Published:** 2021-09-02

**Authors:** Sima Zach, Javier Fernandez-Rio, Aviva Zeev, Miki Ophir, Sigal Eilat-Adar

**Affiliations:** 1 The Academic College at Wingate, Wingate Institute, 4290200 Netanya, Israel; 2 Universidad de Oviedo, Oviedo, Spain

**Keywords:** Exercise behavior, Feelings, Lockdown, Depressive symptoms, COVID-19 global crisis

## Abstract

**Objectives:**

This study aimed at exploring the relationships between physical activity, weight control, and psycho-social aspects of the COVID-19 lockdown, which have characterized the Israeli population’s behavior during the COVID-19 global crisis.

**Design:**

Cross-sectional survey research.

**Methods:**

Participants included 1855 men and women aged 18 and above, from different regions in the country and representing different sectors. They were recruited through the social media in a “snowball” sampling, and filled out a self-administered six-part survey: Demographic background, the International Physical Activity Questionnaire (IPAQ), the positive and negative affect scales (PANAS), the Conor and Davidson resilience scale, a questionnaire for measuring depressive symptoms, and questions regarding weight change based on the Israeli National Health and Nutrition (MABAT) survey.

**Results:**

Routine physical activity (PA) was reported by 76.3% of the participants before the lockdown, 19.3% stopped exercising during this period, and 9.3% began exercising during the lockdown. The participants who were physically active during the lockdown period reported a higher level of resilience and positive feelings, and a lower level of depression, compared with those who were not physically active.

People who were physically active during the lockdown maintained their weight compared with those who were inactive. Concerning weight change, 44.8% of the respondents maintained their weight, and a higher percentage of people reported weight gain than those who reported weight loss.

**Conclusions:**

Continuous PA before and during the COVID-19 lockdown were associated with higher resilience and positive emotions, and depressive symptoms, in people aged 18 and above. Although a causal link cannot be established, in light of the results of the present study, encouraging physical activity may contribute to improving mental health and a sense of self-efficacy, as well as to maintaining weight during a crisis.

## Introduction

The COVID-19 epidemic has become a pandemic and a global health threat. Within a year of the outbreak, COVID-19 infected over 120 million persons worldwide, with more than 2,671,764 confirmed deaths [1]. In Israel, according to the last update of the Israel Ministry of Health (March 17, 2021, 08:49 AM) [2], a total of 823,314 had tested positive, and 6051 deaths occurred [2]. The sudden and rapid spread of the COVID-19 pandemic has shattered the normality of daily life worldwide, and will surely have a long-term impact. On March 15th, 2020, a lockdown was imposed throughout the country. The entire K-12 education system and many jobs transitioned to online. Public places, restaurants, gyms, theaters, community centers, parks, and businesses were shut down to ensure social distancing, limit the movement of the population, and mitigate the effect of the pandemic. People could leave their home only if they were required as essential workers or needed to carry out necessary activities such as purchasing medicines or food. Although this strategy was reported to be efficient for containing the COVID-19 outbreak, the quarantine was also reported as being associated with harmful implications to society.

Lockdown is usually a debilitating experience. The loss of freedom, financial cost, uncertainty over disease status, separation from loved ones, boredom, frustration, and inadequate information may create dramatic harmful effects [3]. Negative outcomes, such as suicide [4], the generation of substantial anger [5], as well as an increase in violence and aggressiveness within the family [6], are just a few examples that have been recorded. Results of a review [3] of 24 articles concerning the short- and the long-term impact of quarantine on psychological well-being, demonstrated that prolonged self-isolation has a negative impact on psychological responses, promoting post-traumatic stress symptoms, confusion, and anger. In addition, quarantine might lead to physical inactivity, which should be of major concern as a great deal of evidence has shown that lack of physical activity (PA) contributes to adverse health changes, such as affecting the immune system [7–9], depression [10], obesity [11], and sleeping problems [12]. Therefore, the potential benefits of a mandatory mass lockdown need to be weighed carefully against the possible psychological costs [3].

Within several months into the pandemic, a myriad of research had already been conducted in many countries regarding its implications on various aspects related to health and lifestyle. Meyer et al. [13], for example, conducted a cross-sectional study with 3052 US adults from all 50 states. Participants self-reported pre- and post-COVID-19 levels of moderate and vigorous PA, mental health and social aspects. Findings showed that PA was lower post-COVID among participants reporting being previously active, but largely unchanged among previously inactive participants. Lack of PA was associated with worsening mental health. Rhodes et al. [14] examined weekly moderate to vigorous physical activity (MVPA) frequency and duration of pre and post COVID-19 restrictions among 1055 Canadian participants aged 18+ years, and related psychosocial and environmental factors. Their findings revealed that participants had decreased weekly MVPA. Maher et al. [15] reported similar results among college students. They found that total minutes of PA were positively associated with positive affect before and during COVID-19 stay-at-home orders. Werneck et al. [16] focused their research on the association between depression and changes in PA and diet behaviors among 41,923 Brazilian adults. Their findings showed participants with a previous diagnosis of depression were at risk for incidence of unhealthy diet behaviors. Wang et al. [17] explored how COVID-19 affected the health-related quality of life (QoL) among 2289 Chinese adults who had been isolated at home for an average of 77 days. More than 50% of the respondents reported that their time engaged in daily PA decreased. During home isolation, 75.2% of the adults rated their sleep quality as very good, and 65% reported that they were satisfied with their QoL.

In Israel, research has also been conducted regarding the relationship between PA and health behavior during the period of The COVID-19 pandemic [e.g., 18, 19, 20]. For example, Dor-Haim and his colleagues [18] surveyed 1202 trainees who exercised on a regular basis. They found that 70% trained less during the pandemic period, and 55% gained weight with an average increase of 1.2 kg. However, those who exhibited a higher physical activity level gained less weight. The Israeli Center for Disease Control [19] conducted a telephone survey with a representative sample of 2580 Israeli participants aged 21+ to examine the changes that have taken place in the use of medical services, and in their mental state, healthy behaviors, and nutrition. In addition, it checked citizens’ compliance with government guidelines from the onset of the plague to the quarantine following the second wave of the plague. They reported that 56.8% of the respondents regularly engaged in physical activity before the Corona plague. Of all those who reported regular physical activity, 65.7% reported that their habits had changed since the Corona pandemic. Of the respondents who reported a change in physical activity habits, 62.1% reported a decrease in the frequency or duration of their activity, and 46.7% reported a decrease in intensity. As for weight control, 28.6% of respondents reported gaining weight since the onset of the Corona plague and 24.1% reported eating more. Lastly, Zach et al. [20] examined the differences between Israeli adults in the age group 70+ and two other age groups (45–59 and 60–69) concerning their healthy and active lifestyle. They surveyed 1202 people and found that in adults at 70+, the physical activity level, physical activity before and during the lockdown, emotions, sleeping hours, and weight change were similar to the other adult groups that were examined (45–59 and 60–69). However, in the older adults groups (70+ and 60–69), resilience and depression symptoms were lower than in the youngest age group.

Considering the findings that emerged from these studies, the Chinese [17] study showed that the majority of the participants reported life satisfaction despite a long duration of isolation; the Brazilian study [16] focused on the association between PA, unhealthy diet behaviors, and depression; the Canadian study [14] focused on PA behavior change; and, the study conducted in the US [13] measured psychological, social, and PA behavior changes pre and post the COVID-19. Differences in the research goals of these studies might be related to cultural differences among the countries. We were interested in finding out similarities as well as the uniqueness of the Israeli population related to other populations that were reviewed.

Israel is a sunny country with some of the characteristics of a Mediterranean lifestyle, such as strong community life and cultural traditions [21, 22]. Greater community resilience – social, economic, infrastructural, and institutional, in addition to higher sunlight exposure, were associated with lower factors of mental health hazards [23]. The first lockdown’s forced isolation and lower exposure to community life and to sunlight were both expected to be risk factors for a decrease in mental health aspects for Israelis – probably more than in other populations. Hence, the present study aimed to examine health behavior changes among Israeli participants. Specifically, we wanted to examine changes in the PA levels during the first lockdown imposed on the Israeli population during the COVID-19 pandemic, and to determine their relationships with weight control and psychological health.

## Methods

We recruited people aged 18 or above living in Israel during the COVID-19 outbreak via different social networks such as e-mail, WhatsApp, Twitter, Instagram, and Facebook). A nonprobability snowball sampling strategy was used. It has been shown that the response rate to online snowball surveys is higher than when other strategies are used [24]. Participants were 1855 people, 566 males and 1289 females, aged 18–90, from all over the country, representing its seven main regions. Participants reported their weight and height, and body mass index (BMI) was calculated by the researchers.

A six-part survey was used, including:

- Demographic background.
- *The International Physical Activity Questionnaire (IPAQ)* [25] – the short version, relating to PA that was conducted during the previous week. Participants had to describe their level of PA, and the frequency, duration, and intensity (see the original instrument for further details). Reliability for the Hebrew version in terms of Alpha Cronbach ranged from 0.59 to 0.90 [26].
- *Positive and Negative Affect Schedule –* PANAS [27]. A 20-item questionnaire assessed positive affects (10 items; e.g. strong, inspired) and negative affects (10 items; e.g. worried, guilty) experienced by the participants in the previous month. Participants rated their feelings on a 5-point scale (1 = hardly at all or not at all; 5 = to a great extent). Reliabilities, in terms of Cronbach alphas, for the original scale were .89 for positive affect and .92 for negative affect [26]. Alphas for the Hebrew version of this scale were used in many previous studies (e.g. [27]) ranged from .80 to .91. In the current study Cronbach alphas were .83 for positive affect and .86 for negative affect.
- *The Connor and Davidson Resilience Scale* [28]

The Connor-Davidson Resilience scale (CD-RISC) is comprised of 25 items, each rated on a 5-point scale (0–4), with a higher score reflecting greater resilience. Internal consistency of the original validation study was .89, and test-retest reliability demonstrated a high level of agreement between the two tests, with an intraclass correlation coefficient of .87. Factor analysis yielded the following five factors: (1) Personal competence, high standards, and tenacity; (2) Trust in one’s instincts, tolerance of negative affect, and strengthening effects of stress; (3) Positive acceptance of change, and secure relationships; (4) Control; and (5) Spiritual influences. As mentioned above, in the current study we conducted factor analysis and came up with two factors: (1) Personal competence and self-control, and (2) Positive acceptance of change. Internal consistency ranged from .87 to .90.

(e) *A questionnaire for measuring depressive moods* [29]

Six questions on a 4-point scale measured depressive symptoms. Internal consistency for the current sample was .86.

(f) Weight control is the term used to discuss managing and maintaining a healthy body weight. Questions regarding weight control were based on the Israeli National Health and Nutrition (MABAT) survey, questions 50–54 [30]: “What is your current weight (without shoes)?”, and “what is your current height? (in cm without shoes)”; the question “did your weight change during the last 2 years?” was changed to “did your weight change since the enforcement of social isolation until the end of the complete lockdown?”

Confirmatory Factor analysis was conducted to reconfirm the following behavior measurement factors for the current cohort: Positive and Negative Affect Schedule – PANAS; The Connor and Davidson Resilience Scale; and a questionnaire for measuring depressive moods. The number of factors to retain was calculated using the Kaiser-Guttman rule (eigenvalue > 1) and the scree plot. Two factors were created for the resilience scale, one factor for the depression scale, and two factors for the PANAS scale with eigenvalues values > 1.

The procedure of the questionnaire’s translation followed the recommendations of other cross-cultural researchers (e.g., [31–35]). All parts of the questionnaire were translated into Hebrew by back-translation and the committee approach. A group of three bilingual translators translated the questionnaire from the language of origin into the target language, and back again. Then errors in translation were corrected by the translating team. Following the translation, a committee discussed the final version of the questionnaire until full agreement was achieved.

Data were collected during the complete lockdown between the dates April 8, 2020 to April 18, 2020. The survey was approved by the Institutional Review Board (IRB), permission [No. 250]. The questionnaire was distributed via e-mail, WhatsApp, Twitter and Facebook to a first wave of participants (the research team’s personal and professional contacts). They were asked to share the link with their contacts and ask these to do the same to obtain a wider and less biased sample.

Involvement in PA was defined as an answer to the question: “Were you engaged in physical activity before the lockdown?”, along with the answer to the question: “Are you doing physical activity during the lockdown?”, and categorized into a dichotomous variable: yes or no. Reported adherence to physical activity prior to the pandemic, and during the lockdown, was categorized into four groups: (a) No/No – did not do physical activity before the lockdown/not doing physical activity during the lockdown; (b) No/Yes – did not do physical activity before the lockdown/doing physical activity during the lockdown; (c) Yes/No – did physical activity before the lockdown/not doing physical activity during the lockdown; (d) Yes/Yes – did physical activity before the lockdown/ doing physical activity during the lockdown. The background characteristics of the four groups of physical activity behavior are presented by means and standard deviations for normal variables, and by frequencies for categorical data. One-way ANOVA was conducted to compare the reported psychological variables among the four groups of physical activity behavior, and a Chi Square test was used to compare weight change in those four groups. Multi-Dimensional Scaling Analysis was performed using the ALSCAL procedure in IBM SPSS (Version 25.0).

## Results

The sample includes 1855 participants, 571 males (30/6%), and 135 non-Jewish (7% of the sample). Mean age 50.2 (S.D = 14.5), 5.1% at the age 18–24. 31.6% at the age 24–44, 47%.1 at the age 45–64, and 16.1% at the age 65+. The current sample is not a representative sample of the population. The sample was made up of about 31% men compared to 51% in the population over the age of 18 in Israel; non-Jews constitute 7% of the sample, in contrast to their share in the population of 25.9%. The age distribution is also different: while 18–24 year-olds are 16.5% of the population aged 18 and over, in the sample they are only 5.1%. Ages 25–64 constitute the majority of the sample – 78.7% compared to 72.1% in the Israeli population aged 18 and over. Those aged 65 and over have a higher representation in the sample (16.1%) than their percent in the population (11.4%).

Descriptive statistics of the four groups of PA according to the survey’s background characteristics are presented in Table 1.

**Table 1:** Doing physical activity before/during the lockdown (*N* = 1855)

Doing physical activity* before/during the lockdown	No/No (***n*** = 268)	No/Yes (***n*** = 172)	Yes/No (***n*** = 358)	Yes/Yes (***n*** = 1057)
**Males (%)**	68 (12.0)	41(7.2)	96 (17.0)	361 (63.8)
**Females (%)**	200(15.5)	131(10.2)	262(20.3)	696(54.0)
**Age (years)**	46.9 (13.6)	47.0 (15.1)	51.9 (14.4) ^a,b^	50.9 (14.5) ^a,b^
**BMI (kg/m**^**2**^**)**	27.3 (5.8) ^b,c,d^	25.7 (4.7)	25.7 (4.0) ^d^	24.9 (4.1)
**Weight change (kg)**	1.0 (2.5) ^d^	0.3 (2.1)	0.1 (2.3) ^b,d^	0.3 (1.8)

^a^ Compare with No/No; ^b^ Compare with No/Yes; ^c^ Compare with Yes/No; ^d^ Compare with Yes/Yes

The average number of stay-at-home days was 33.7 (Kg) ± 4.2 (Median 32). Reported weight change was small (0.54 Kg ± 2.06). Most of the participants (76.3%) reported being physically active before the lockdown, while 25% (*n* = 358) of them stopped during the lockdown. Among those who were not active before the lockdown (*n* = 440), 39% (*n* = 172) started PA during the lockdown. Participants who stopped engaging in PA during the lockdown were either slightly or significantly older compared with those who were not active before the lockdown. Those who reported being and staying physically active were also older than those who were not active before the lockdown, and they had a lower BMI compared with those who had not started PA and those who were active but stopped during the lockdown. According to the BMI, people were within the normal to overweight range (with average of normal weight) (25.46 ± 4.47). Only those who reported being inactive before and during the lockdown were on average overweight and had a significantly higher BMI compared with all other groups. Those who were not active before and during the lockdown gained more weight during the lockdown compared with those who were active.

We examined the differences between the four categories of PA according to the following age groups: 18–24, 25–44, 45–64, and 65+. The majority of the sample (78.8%) was in the age range of 25–64. 14.8% of the participants reported being inactive both before and during the lockdown. A small percentage began exercising during the lockdown (9.3%), and more so among the age groups 25–44 and 45–64 than the younger and the older groups (data not shown). At the age range 45–64, 28% of the participants were physically active before the lockdown but stopped during the lockdown, which is significantly different and higher than the other age-range groups, especially compared with 16% at the age-range group 18–24 (*p* < 0.05). As for doing PA during the lockdown and being active before it began, it appears that the majority of the current cohort were active people.

Descriptive statistics for resilience, emotions, and depression symptoms according to the four categories of PA are presented in Table 2. The number of respondents available for analysis in Table 2 includes those who answered the PA, resilience, and emotions questionnaires. There was no significant difference in PA distribution comparing those who replied to all the psychological questions as presented in Table 2 (*n* = 1681), to those who did not answer one or more of the psychological questions in the resilience and emotions questionnaires (*p* = 0.267).

**Table 2:** Resilience, emotions and depression symptom measures according to physical activity before during the lockdown (*N* = 1681)

Exercising	Range of Scores	No/No *(a) ***n*** = 242	No/Yes(b) ***n*** = 159	Yes/No(c) ***n*** = 316	Yes/Yes(d) ***n*** = 964
**Positive acceptance of change**	0–4	2.87 (0.74)	3.00 (0.73)	3.01 (0.69)	3.07 (0.72)* ^a^
**Self-competence and self-control**	0–4	2.91 (0.68)	3.01 (0.74)	3.03 (0.67)	3.10 (0.69) **^a^
**Positive emotions**	1–5	2.56 (0.88)	2.63 (0.83)	2.70 (0.83)	2.84 (0.82) **^a,b^
**Negative emotions**	1–5	1.94 (0.75)	1.92 (0.78)	2.09 (0.84)	1.96 (0.76)
**Depression symptoms**	1–4	2.15 (0.73)**^d^	2.0 (0.64)	2.18 (0.70) **^b,d^	1.98 (0.66)

Resilience is presented on a 5-point scale from 0 to 4. Positive and negative affect schedule runs from 1 to 5. Depression symptoms are presented on a 4-point scale

*^a^ Compared with No/No; ^b^ Compared with No/Yes; ^c^ Compared with Yes/No.; ^d^ Compared with Yes/Yes

** Statistically significant at *p* < 0.05

Overall, in all four groups, positive feelings received higher scores than negative feelings. Participants who reported performing PA both before and during the lockdown had higher scores on positive acceptance of change, self-competence, and self-control compared with those who were not physically active either before or during the lockdown. Their positive acceptance of change was also higher than that of those who were not physically active before, and started PA during the lockdown. There was no difference between the groups in negative feelings.

The lowest depression symptoms score was demonstrated for those who were physically active before and during the lockdown. The second lowest score was for those who started PA during the lockdown (compared to those who were doing PA and stopped), while the group of participants who were not active before and during the lockdown had the highest level of depression.

Weight change was categorized into seven categories in the questionnaire (three of gaining weight, three of losing weight, and one of no change). However, due to the small number of participants in the extreme groups (losing or gaining over 5 kg), the categories were merged into five groups, as follows: (a) (− 10)-(− 2) [11%], (b) (− 2)-(− 0.5) [29.4%], (c) No change – (− 0.5)-(0.5) [41.8%], (d) 0.5–2 [12.5%], and (e) 2–10 [5.0%]kg. We examined the differences between the four categories of PA according to these five groups. About one-third to one-half of the sample had no weight change. However, a significantly higher percentage of people who were physically active during the lockdown maintained their weight compared with those who were inactive (*p* < 0.05). About 25–36% gained 0.5-2 kg, a higher percentage of those participants being inactive during the lockdown vs. active participants. A Chi Square test revealed significant higher weight gain among both groups who were not active during the lockdown (No/No; Yes/No) compared to those who were active during the lockdown (No/Yes; Yes/yes). More specifically: Yes/No compared with No/No (*p* < 0.001), Yes/No compared with No/Yes (*p* < 0.01), and Yes/Yes compared with Yes/No (*p* < 0.001).

**Fig. 1:**
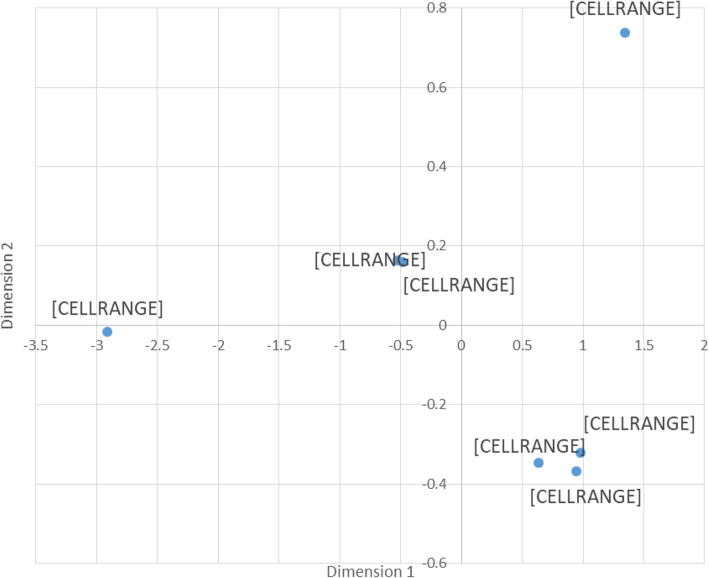
Multi-dimensional scaling of the Euclidian distance between the research variables

The research integrates three domains: PA behavior, psycho-social aspects, and weight control during the lockdown. Therefore, a Multi-dimensional Clustering was performed (see Fig. 1) using the Euclidian distance between the variables.

Figure 1 illustrates the group composite stimulus map summarizing the participants’ scores on the three domains that were surveyed. As shown, the negative emotions (PANAS negative and depression symptoms) are not far from the origin and they are close to each other. Weight change is placed on the left quadrant, negative side, near the X axis, while PA is placed in the right quadrant, positive region of the Y axis. The positive emotions are assembled together near the negative Y axis. In other words, the location of the negative emotions is close to the beginning of the axes (0,0), meaning that they are quite low, but negative in relation to the other variables. On the other hand, the positive emotions are in the positive part of the X-axis but in the negative part of the Y-axis. That is, they have an opposite element to the negative (diagonally).

## Discussion

In the current study we aimed at exploring the relationships between PA, psycho-social aspects, and weight control that characterized the current study population’s behavior during the COVID-19 global crisis. The central focus of our survey was health behavior. More specifically, the focus was on PA habits. Two major results may have long-term implications. The first is that physically active people – whether or not they were physically active before the lockdown – reported a lower level of depression symptoms compared with inactive people. This finding is along the lines of others who demonstrated the relationships between depression symptoms and PA (e.g. in a review by Kandola and colleagues [36]), in the elderly (e.g. [37]), in adults (e.g. [10, 16, 20]), and in adolescence and childhood (e.g. [38]). Moreover, our findings showed that not only was inactivity associated with depression, but changes in PA habits from being active pre-lockdown to becoming in-active during lockdown were related to increased depressive symptoms. Others [13, 14] found similar results in their studies, in which they examined the level of MVPA pre-COVID-19 to post COVID-19 and the associated depression. Both studies found that a decrease in MVPA is related to an increase in depression.

Second, active people reported on having more positive emotions on. Since the lockdown was found to be related to risk behavior, such as suicide [3], anger [4], and violence and aggressiveness [6], we expected the participants to report negative emotions during this period of time. However, active people differed significantly from inactive people: they reported on a significantly higher level of positive emotions compared with their counterparts who were not active. As others have asserted that the psychological construct of well-being includes both aspects – low rates of negative emotions along with high rates of positive ones (e.g. [13, 15, 38, 39]), our results suggest that being active is associated with a better reported well-being compared with being inactive.

These results suggest that PA is a means for managing a healthy lifestyle – both physically and mentally, not only during routine times but also in a global, ongoing crisis atmosphere that creates pressure and uncertainty, and that decreases the likelihood of people to be at risk for developing negative feelings and behaviors (e.g. [6, 9, 11, 13, 17]).

As for the association between PA and weight, the majority of the participants maintained their original weight. Those who were active before the lockdown and continued to be active during the lockdown gained less weight than those who were not active during the lockdown, regardless of whether they were active before or not. In addition, participants who stopped exercising during the lockdown were older than those who continued to exercise, and their BMI was higher. Nevertheless, those who continued to exercise were older than those who did not exercise before the lockdown, and weighed less. The current results imply that active people have a regular set of behaviors, and even when facing the restrictions placed by a lockdown they do not change their healthy habits. Nevertheless, a recent publication from Israel concerning PA and healthy behavior during the COVID-19 pandemic found that.

65.7% of those who reported being regularly active before the pandemic, reduced their level of PA. Among them, 59% reported not returning to their usual PA after the second lockdown [19]. As for weight changes, our results goes along this report, meaning that active people gained less weight. Along this line, Werneck et al. [16] found that active participants specifically demonstrated a higher frequency of vegetable or fruit consumption, and less frequency of ultra-processed food consumption. These two factors –weight control and PA together, probably contributed to positive psychological functioning: a high level of resilience, a high level of positive emotions, and a low level of depression.

Some limitations should be addressed. Firstly, the current sample is not representative of the Israeli population, due to the “snowball” sampling strategy. The selection bias is also evident with the high percentage (56%) of those reporting to be physically active (yes/yes). Since the authors are from a sports college, it is probable that the questionnaire arrived to more – but not exclusively – physically active people. Still, another Israeli study with a representative sample of the adult population aged 21+ [18, 19] reported on similar rates of PA engagement. Secondly, it is a cross-sectional survey and therefore causal associations cannot be addressed. Thirdly, we should consider the meaning of over representation of female respondents on the study variables and the study implications. Lastly, the range of ages was centered mainly on two age groups; other age groups are not well represented.

## Conclusions

Continuous PA before and during the COVID-19 lockdown was associated with higher resilience and positive emotions, and low depressive symptoms. It should be noted that continuous PA before the lockdown might be interpreted that participants continued their habits only until the lockdown, while the study findings point to the unique combination of before and during (yes/yes).

These results have several implications:

The first lockdown during the COVID-19 pandemic period was an opportunity to assess the mental importance of exercising during a crisis. After the first lockdown additional lockdowns were imposed, and the economic crisis following COVID-19 is expected to remain, even in the period after the epidemic is eradicated. In light of the results of the present study, encouraging physical activity may contribute to improving mental health and a sense of self-efficacy, as well as contributing to maintaining weight during a crisis. Although a causal association cannot be established, we recommend that strategies be developed to enhance free PA in any future disasters/emergencies/catastrophic times.

Some possible techniques are: using available free facilities in the public space (urban parks, gym equipment in outdoor public areas, etc.) and the private space (via the Internet). During lockdowns people should be allowed to carry out exercise in open space. Some of these conclusions were implemented in the second lockdown, in which people were allowed to exercise in open space. Organizations, systems and institutions such as the Ministry of Health, the health system, and the education system, together with employees, teachers and educators, should not only share these messages but also organize demonstrations of a variety of exercises for the population via the mass and social media. We join Neiman [8], who stated that the COVID-19 pandemic is a wake-up call – a tocsin – to the world, in his conviction that primary prevention focused on health behaviors, including PA and weight control, is crucial.
