# Correction to: Physical activity, resilience, emotions, moods, and weight control, during the COVID-19 global crisis

**DOI:** 10.1186/s13584-021-00489-3

**Published:** 2021-09-15

**Authors:** Sima Zach, Javier Fernandez-Rio, Aviva Zeev, Miki Ophir, Sigal Eilat-Adar

**Affiliations:** 1 The Academic College at Wingate, Wingate Institute, 4290200 Netanya, Israel; 2 Universidad de Oviedo, Oviedo, Spain

## Correction to: Israel Journal of Health Policy Research (2021) 10:52 https://doi.org/10.1186/s13584-021-00473-x

Following publication of the original article [1], the authors identified that an incorrect version of Fig. 1 was published.

The incorrect (Fig. 1) and correct (Fig. 2) version of Fig. 1 are published in this correction article. The original article has been updated.

**Fig. 1:**
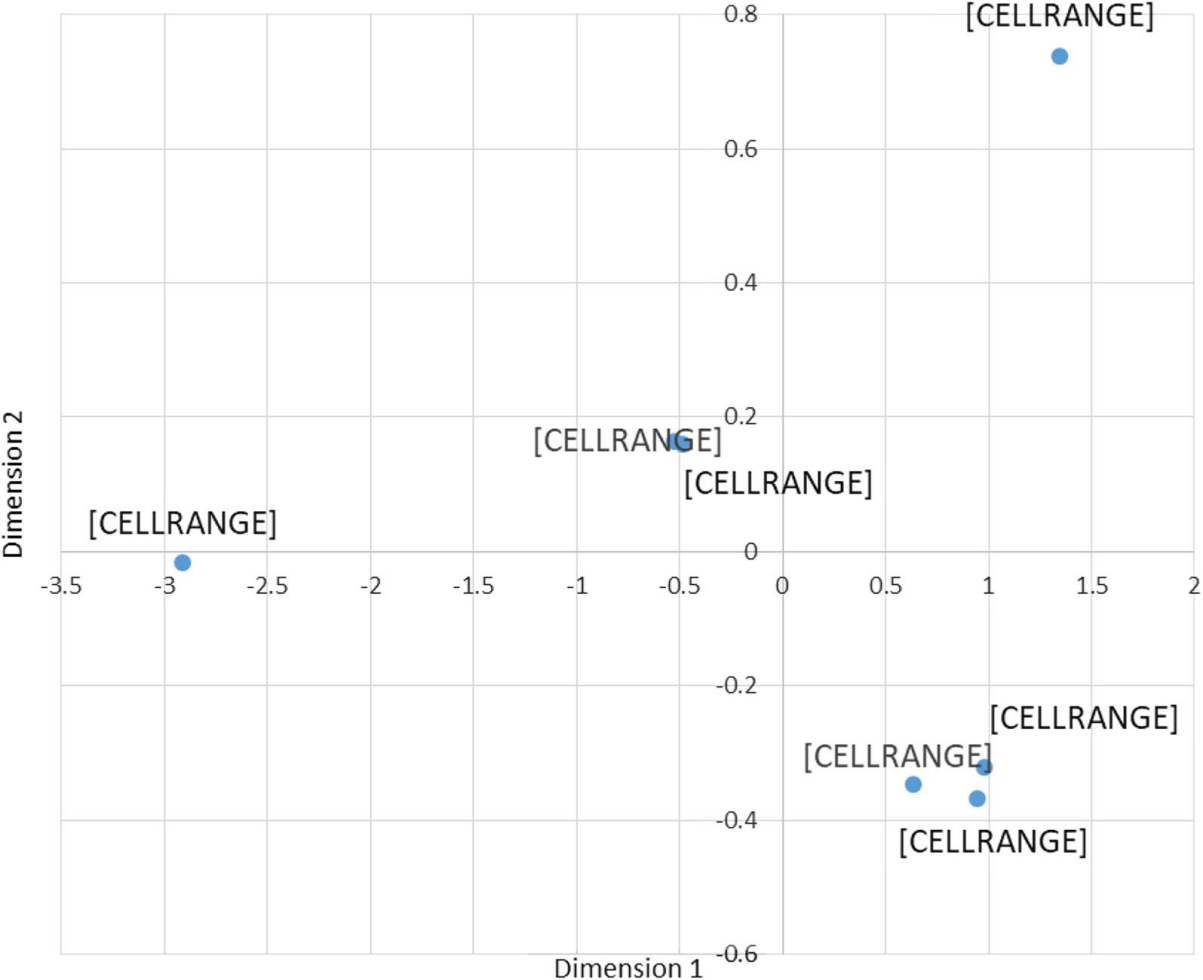
Incorrect version of Fig. 1 as originally published

**Fig. 2:**
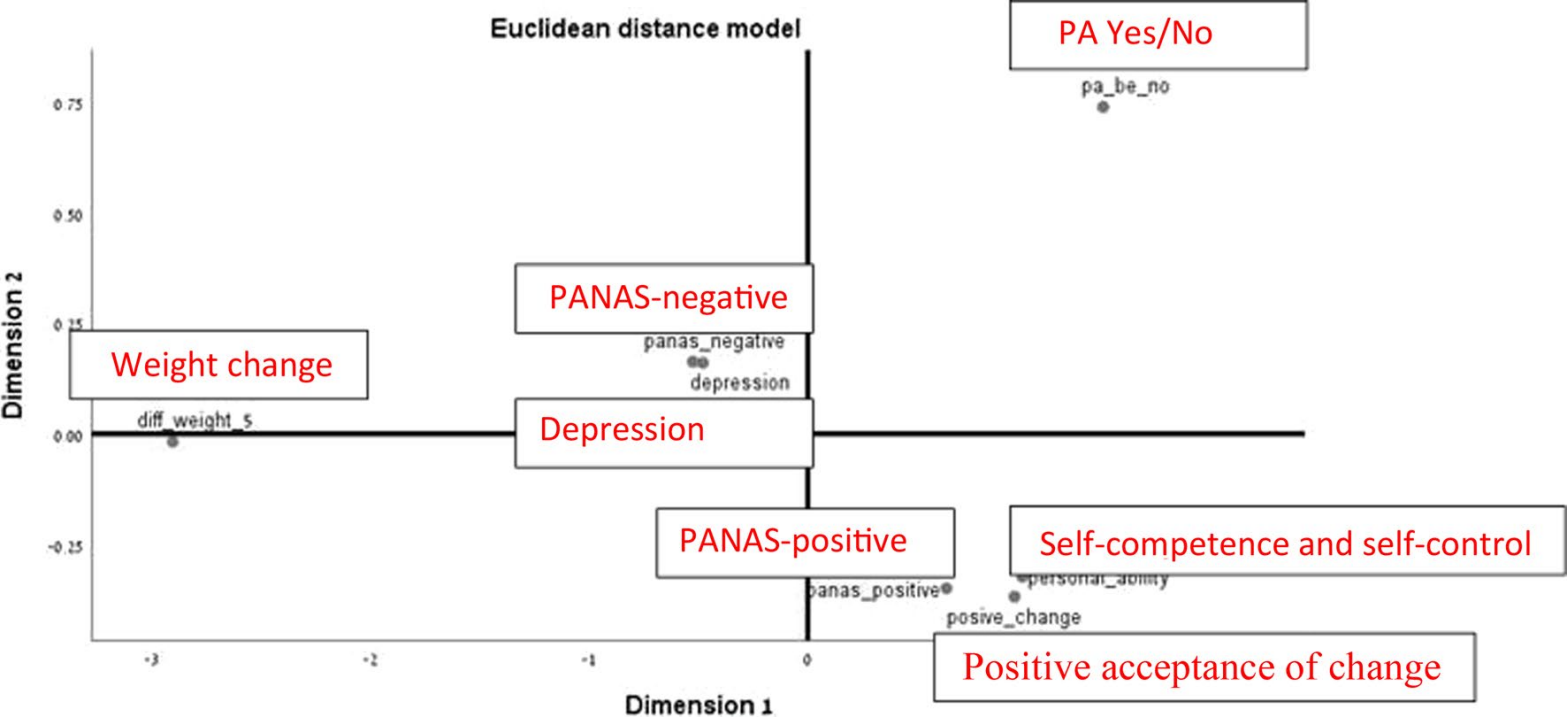
Correct version of Fig. 1
